# Revealing the Adverse Impact of Additive Carbon Material on Microorganisms and Its Implications for Biogas Yields: A Critical Review

**DOI:** 10.3390/ma16237250

**Published:** 2023-11-21

**Authors:** Michał Kozłowski, Chinenye Adaobi Igwegbe, Agata Tarczyńska, Andrzej Białowiec

**Affiliations:** 1 Department of Applied Bioeconomy, Wrocław University of Environmental and Life Sciences, 51-630 Wrocław, Poland; michal.kozlowski@upwr.edu.pl (M.K.); or ca.igwegbe@unizik.edu.ng (C.A.I.); tarczynskaagata7@gmail.com (A.T.); 2 Department of Chemical Engineering, Nnamdi Azikiwe University, P.M.B. 5025, Awka 420218, Nigeria

**Keywords:** biochar, hydrochar, biogas, anaerobic digestion, carbon material

## Abstract

Biochar could be a brilliant additive supporting the anaerobic fermentation process. However, it should be taken into account that in some cases it could also be harmful to microorganisms responsible for biogas production. The negative impact of carbon materials could be a result of an overdose of biochar, high biochar pH, increased arsenic mobility in the methane fermentation solution caused by the carbon material, and low porosity of some carbon materials for microorganisms. Moreover, when biochar is affected by an anaerobic digest solution, it could reduce the biodiversity of microorganisms. The purpose of the article is not to reject the idea of biochar additives to increase the efficiency of biogas production, but to draw attention to the properties and ways of adding these materials that could reduce biogas production. These findings have practical relevance for organizations seeking to implement such systems in industrial or local-scale biogas plants and provide valuable insights for future research. Needless to say, this study will also support the implementation of biogas technologies and waste management in implementing the idea of a circular economy, further emphasizing the significance of the research.

## 1. Introduction

The energy demand has been rising significantly since the Industrial Revolution, and our civilization still needs energy to develop and keep a high living standard. The energy demand may increase in the future, but it highly depends on social trends [1]. Needless to say, even if worldwide society reduces energy consumption, they still need to provide energy to prosper [2].

Generally, since the Industrial Revolution, fossil fuels have started to be the main sources of energy for human civilization [3,4]. Despite the wide use of these fuels and the fact that thanks to the energy obtained from this type of fuel, humanity could afford intensive development [5], they also have a negative impact on our environment and are one of the main factors causing global warming [6,7].

To meet the requirements of global warming and to secure future generations with sound and sustainable sources of energy, the reliance on fossil fuels must be reduced [8,9]. The use of finite resources in energy production puts energetic security at stake, making it dependent on mining and oil/gas extraction (non-renewable energy sources) and sometimes external supply chains (international export of strategic energetic resources). Moreover, the influence of fossil fuel combustion has proven perilous for the environment [10,11]. Those aspects form a pressing need to provide energetic security and implement renewable energy resources to a further extent [12]. 

One of the most promising concepts for a reduction in the reliance on fossil fuels is renewable natural gas (later mentioned as RNG) [13,14], which can not only be included as a carbon-neutral source of energy but can be treated as an integrated part of the circular economy [15,16,17]. However, some of the residual waste, i.e., sawdust from sawmills [18] or wheat straw [19], can be used as a combustible fuel, and the waste itself can be transformed into additives for fuel production. There is a substantial number of studies treating the issue concerning biomass carbon additives and their influence on the anaerobic digestion process [9,20,21]. Although they can catalyze and augment fuel production to a certain extent, biogas production is far more perplexing, and every single aspect must be analyzed explicitly.

Biogas is known as a product of anaerobic digestion, where microorganisms biodegrade organic substrates under anaerobic conditions and biomass is transformed during methane fermentation [12]. The process can be divided into four phases: hydrolysis, acidogenesis, acetogenesis, and methanogenesis [22,23]. 

Needless to say, each of these phases has a crucial role during anaerobic digestion. Thus, it is worth briefly describing every phase. Hydrolysis is an initial phase during the decomposition of organic matter; microorganisms use their enzymes to decompose macromolecules into smaller components, like carbohydrates, lipids, and proteins that are assimilable for the acidogenic microbiota. In the second phase of acidogenesis, microbiocenosis, using the products obtained in the previous phase, synthesizes volatile fatty acids and alcohols. The third phase is organogenesis, where the main process is the conversion of higher volatile fatty acids and some other products obtained in the earlier phases to acetate. Moreover, hydrogen is also synthesized during this phase. The final phase of biomethane production is methanogenesis. In this particular phase, we can divide it into two paths of methane formation [24,25]. The first is hydrogenotrophic methanogenesis, during which hydrogen and carbon dioxide are used to synthesize methane [26]. The second path is the decomposition of acetate to methane and carbon dioxide [27].

It is worth mentioning that the biogas production itself could also be dependent on the AD treatment technology used; for example, the Up-flow anaerobic sludge blanket technology can create better living conditions for *Desulfobacterota* microorganisms, which can compete with methanogenic microbiocenosis for hydrogen and acetate, causing a decrease in the efficiency of methane synthesis [28]. 

Biogas consists of various gasses, mainly methane, evaporated water, carbon dioxide, and hydrogen sulfide. Since 2010, there has been a 4% increase in biomethane production potential, and waste-derived fuels have gained much interest [8]. Over the last few years, there have been multiple attempts to improve the processes of biogas production including the use of biochar [29]. Moreover, digestate recirculation could also have a positive impact on improving biogas yields [30].

When people hear about using biochar in energetics, quite often their first thought is about using biochar, like normal coal in a coal power plant by combining it; of course, that thought is correct. Biochar has multiple applications in the field of energy generation. It can be utilized as a standalone fuel in power plants, where it demonstrates potential as a greener alternative to conventional coal [31]. Furthermore, when combined with coal, biochar can serve as an additive, enhancing the combustion efficiency and reducing emissions [32]. The heat generated from biochar combustion can be harnessed not only for electricity production but also for various industrial processes, such as iron forges [33]. Additionally, biochar offers a promising avenue as a substitute for pulverized coal [34], contributing to lower greenhouse gas emissions.

Another use of biochar in energetics (and most important in this article) is its use as a catalyst in biogas production, where it plays a crucial role in anaerobic digestion processes and microbial activities [35]. This aspect will be explored in detail in the subsequent sections. 

### 1.1. Background Information on Carbon Materials and Their Role in Biogas Production

Biochar is a material produced from organic matter, i.e., cellulose, lignin, and proteins, during the pyrolysis process. Biochar production could be a brilliant way to utilize biowaste and make that material useful, for example, could be used in agriculture and water purification [36], and the most important topic in this article is bioenergetics [37]. Needless to say that biochar additives could affect methane fermentation solution stability, mainly to increase direct interspecies electron transfer (DIET) [38] and buffer capacity [39], and it can be highly affected by microorganisms. Biochar additives could be an abundant source of microelements and improve features of methane fermentation solutions, being a habitat of microlife [40,41]. But, on the other hand, the biochar influence could be also harmful [42,43]; therefore, the authors of this publication will focus on a critical view of biochar additives to the methane fermentation solution.

### 1.2. Importance of Understanding Carbon Material Molecular Properties for Optimizing Biogas Yields

Biogas plants could be an excellent part of the renewable energy mix in the worldwide energy system [44], mostly in the regions abundant in agricultural residues and organic waste [45]. Biogas could be used to produce not only electricity but also heat [46]. This is because the optimization of biogas production yields is an important case in the implementation of a renewable energy energetic mix. Moreover, the technique of optimizing biogas production should not only be cost-efficient, but also its production should have as little impact on the environment as possible, considering that it is promising to investigate the influence of biochar to enhance biogas production. 

### 1.3. Purpose of the Critical Review

The beneficial influence of biochar on microbiocenosis in methane fermentation solutions has been proven in many publications [47,48,49] and experiments, but some articles bring some information about the possibilities of a negative influence [50,51,52]. The importance of this kind of critical review could be precious not only to properly understand the mechanism of the influence of biochar on microorganisms but also could be useful for industrial purposes. For example, information about the possibilities of a harmful influence with the specific cause could help to avoid some mistakes during the inclusion of biochar additives in biogas plants on an industrial scale.

## 2. Overview of Biochar Molecular Properties

### 2.1. Definition and Classification of Carbon Materials

Carbon is a material composed of carbon atoms and is known for its unique properties, such as high electrical conductivity, as well as high strength resistance to chemicals [53,54]. Carbon materials are a diverse class of materials with various properties and applications. They differ in their physicochemical characteristics, such as pore size and shape, surface area, size, volume of pores, functionality of the surface, and chemical inertness. Carbon-based materials for environmental applications may be classified into activated carbon, graphitic carbon (including carbon nanodots, carbon nanotubes, graphene, and fullerenes), biochar, carbon black, carbon-based nanomaterials (including carbon nanoparticles, carbon nanotubes, fullerenes, and graphene), and carbon composite. Activated carbon (amorphous carbon, including carbon black) is a type of carbon material that has a disordered atomic arrangement, which makes it non-crystalline, and is used in various applications, including adsorption [55,56,57], gas storage [58,59], biogas production [60,61], and catalyst support [62,63], due to its high reactivity. Activated carbon is a highly porous form of carbon that is produced by treating carbon-rich materials, such as coconut shells, wood, or coal, with steam or chemicals to create a network of small pores and a large surface area. Similarly, biochar is also classified under the category of amorphous or non-graphitic carbon due to its disordered and non-crystalline structure [64,65]. Biochars are produced through the pyrolysis of organic materials, typically plant biomass, in a low-oxygen environment [66]. Graphitic carbon is a type of carbon material that has a layered atomic arrangement, with strong covalent bonds within each layer and weak van der Waals forces between the layers [67,68]. Graphitic carbon can exist in different forms, such as graphite, graphene, carbon nanodots, and fullerenes and carbon nanotubes [69,70]. Graphitic carbon materials are used in various applications, including electronic devices, energy storage, and reinforcement in composites [71], because of their high electrical conductivity, high specific strength, and resistance to environmental degradation/oxidation [72]. Carbon nanotubes are cage-like carbon molecules with unique electronic/mechanical/thermal properties and excellent biocompatibility [73]; they are used in various applications, such as drug delivery, nanoelectronics, cancer treatment, and tissue engineering [74]. Also, we have carbon composites that combine carbon fibers or other carbon materials with a matrix material, such as an epoxy resin. Carbon black is a finely divided form of carbon with a high surface area and particle size, usually employed as a reinforcing filler in rubber and plastic products (composite preparation), as well as in inks and coatings [75]. Carbon-based nanomaterials include a broader range of carbon-based particles, which can encompass both graphitic and non-graphitic structures at the nanoscale [76]; these materials have gained significant attention due to their unique electronic, mechanical, and thermal properties, leading to applications in fields, such as electronics, materials science, medicine, and energy storage [77]. Carbon nanodots are tiny carbon-based structures, usually less than 10 nanometers in size, that exhibit quantum confinement effects [78]; they are often used in various applications, including photocatalysis, bioimaging, and sensing [79]. Fullerenes and their derivatives represent the initial class of carbon-based nanostructures to be identified [80]. Graphene is a single layer of carbon atoms arranged in a hexagonal lattice [81]. Graphene is considered a “wonder material” due to its unique and promising characteristics, including high electrical and thermal conductivity, mechanical strength, and flexibility [82]. Graphene has wide-ranging applications in electronics, energy, materials, and medicine [83]. Carbon composites have high strength, thermal stability, and stiffness [84] and are used in various applications, such as aerospace, sports equipment, energy storage, and automotive industries [85]. The versatility of carbon materials makes them one of the most important materials in modern science and technology [86,87].

### 2.2. Overview of the Properties of Biochar 

Biochar is a charcoal-like substance that is produced by heating biomass, such as wood, organic biomass, agricultural waste or crop residues, manure, and sludge, in the absence of oxygen [88,89]. The process, called pyrolysis, converts the biomass into a carbon-rich material. Biochar has gained significant attention in recent years due to its potential uses, including improvements in soil fertility [90], carbon sequestration [91], energy production [92], water treatment [93], livestock feed [94], biogas production [21], and building materials [95]. However, more research is needed to fully realize the potential of biochar in these and other applications. 

The most important properties of carbon materials that make them good for application in many processes include surface area, porosity, stability, functional groups, low bulk density, high carbon content, particle size, higher cation exchange capacity (CEC), and conductivity. Biochar has a highly porous structure, which makes it an excellent material for water and nutrient retention [96]. The pores also provide a habitat for microorganisms, which can improve soil health. The particle size can affect the biochar’s porosity and surface area, as well as its ability to retain water and nutrients [97,98,99]. Biochar has a large surface area, which can adsorb nutrients, pollutants, and organic compounds from the soil, air, and water [100,101]. Biochar typically has a slightly alkaline pH, which can help neutralize acidic soils and improve nutrient availability [96]. Biochar can contain various plant nutrients, such as nitrogen, phosphorus, and potassium, depending on the feedstock and pyrolysis conditions. The high carbon content (65% to 90%) makes biochar a stable carbon sink, which can sequester carbon, treat water, etc. [102]. Biochar can have a high cation exchange capacity (CEC) [103,104], which means (i) it can attract and retain cations, such as calcium, magnesium, and potassium, in the soil [103,105] and (ii) bind some pollutants, including carbon dioxide and heavy metals, by exchanging cations [106]. Biochar can be stable for long periods, but its stability and other properties can also depend on the feedstock, biomass pretreatment, and pyrolysis conditions, such as the carbonization temperature and residence time [107,108]. Biochar produced at a high pyrolysis temperature has low values of CEC and contents of volatile matter, but a high specific surface area, porosity, pH, and ash and carbon content [89,104,109,110]. The breakdown of organic materials on a massive scale is probably accountable for this [89]. Also, Tomczyk et al. [89] found that even at higher pyrolysis temperatures, biochars made from animal litter and solid waste feedstocks have decreased surface areas, carbon contents, and volatile matter and greater CEC than biochars made from crop residue and wood biomass. The reasons for this disparity include wide variations in the biomass’s lignin, cellulose, and water content. Higher temperatures during pyrolysis are linked to an increase in the ash content and oxygen functional groups [89,111]. Shariff et al. [112] observed that the production of biochar will be increased with increased lignin content in feedstock. The higher lignin content in plant biomass has been reported to promote carbonization and increase biochar carbon content and ash content [89,113].

Overall, the high porosity, stable carbon content, and renewable energy potential of biochar make it a promising material for improving the performance of anaerobic digestion systems and increasing biogas production.

### 2.3. Method of Biochar Preparation

Biochar has been produced using various techniques. The method of biochar preparation can vary depending on the type of biomass, the scale of production, and the desired properties of the biochar and its usage [114]. Biochar production has been produced by researchers through pyrolysis [115], hydrothermal carbonization [116,117], gasification [118], a microwave method [119], and torrefaction [120]. Pyrolysis is the most commonly used technique for biochar production, which involves the heating of organic materials in the absence of oxygen, thereby converting the organic matter into a stable form of carbon that can be used for different applications, as well as gasses and liquid byproducts. It can be used to convert a wide range of biomass feedstocks, including agricultural residues, forestry waste, and municipal solid waste, into biochar. The temperature of pyrolysis can range from 300 °C to 1000 °C [121], and the process can be carried out in different types of reactors, such as fixed-bed, fluidized bed, and rotary kiln reactors [122]. Hydrothermal carbonization (HTC) involves the conversion of biomass into biochar through the application of high temperature and pressure in the presence of water [123]. The HTC process typically involves heating the biomass in water at temperatures ranging from 180 °C to 250 °C and pressures of 1–5 MP for several hours [124]. However, the process also has some limitations, including the high energy requirements for heating and pressurizing the biomass, which can make the process expensive [125]. Gasification involves heating the feedstock in a gasifier between 500 and 1000 °C [126], which converts the organic matter into a combustible gas called syngas, and leaving behind a solid residue called biochar [127]. However, gasification also has some limitations, including the high capital and operating costs associated with the equipment and the complexity of the process [128]. The microwave-assisted pyrolysis involves heating the feedstock using microwaves in the absence of oxygen, which can rapidly heat the organic matter and produce biochar, including gasses and liquid byproducts. One advantage of microwave-assisted pyrolysis is that it is energy-saving [129]. Torrefaction, also known as mild pyrolysis, is a thermal conversion process that involves heating biomass to moderate temperatures (200–350 °C) in the absence of oxygen, which results in the production of biochar [126,130].

While various methods, including pyrolysis, are employed for biochar production, modeling pyrolysis processes has been a subject of significant interest in the literature. Several studies have delved into mathematical models and simulations to better understand and optimize the pyrolysis of different biomass feedstocks [131]. For instance, works by Barr et al. [132] and Papadikis et al. [133] have explored the intricacies of pyrolysis kinetics and the reactor design. These modeling efforts contribute to enhancing the efficiency and sustainability of biochar production processes.

### 2.4. Relationship between Biochar Properties and Their Impact on Biogas Yields

The properties of biochar impact biogas yields. Biochar acts as a substrate for the growth of microorganisms that produce biogas, such as methane [134]. The properties of biochar, such as porosity, surface area, pH, nutrient content, and CEC, can impact biogas yields, as biochar with high values of these properties can enhance biogas production by providing a larger surface for microorganisms to grow, neutralizing acidic conditions, supplying essential nutrients, and attracting and retaining cations [20]. The porosity and surface area of biochar is an important factor that affects the biogas yield. Biochar with high porosity can provide a larger surface area for microorganisms to grow, which can provide a large area for gas production to occur, thereby increasing biogas production [135,136]. The pores can also help to retain moisture and nutrients, which can further enhance the biogas yield. Studies have shown that biochar with a higher mesoporosity can significantly increase biogas production [137,138,139]. In addition, the high surface area of biochar can help to adsorb and reduce the concentration of inhibitory compounds, such as ammonia and volatile fatty acid organic compounds, that can hinder the growth of microorganisms and reduce biogas production [48,50].

The pH of biochar can also impact biogas yields. Biochar with a high pH can help to neutralize acidic conditions in the anaerobic digester, which can improve the growth of biomethane-producing microorganisms [140]. Functional groups in biochar can affect biogas production through their impact on the physical and chemical properties of the biochar. The type and number of functional groups present in the biochar can affect its surface area, porosity, and chemical reactivity, which can influence its ability to act as a substrate for microbial activity in biogas production. Functional groups, such as carboxyl (-COOH), hydroxyl (-OH), and amino (-NH_2_) groups can act as binding sites for nutrients and microorganisms, increasing their availability for biogas production [141,142]. Additionally, functional groups can interact with other components in the digestive system, such as metals and organic compounds, which can affect the efficiency of biogas production [143,144]. Generally, the effect of functional groups on biogas production will depend on the specific type and number of functional groups present in the biochar, as well as the composition of the microbial community involved in the process [35].

Biochar with a high nutrient content, such as nitrogen, phosphorus, and potassium, can provide essential nutrients to the microorganisms, which can enhance the biogas yield [145]. Even the cation exchange capacity (CEC) of biochar can also impact the biogas yield. Biochar with a high CEC can attract and retain cations, such as calcium and magnesium, which can improve the growth of methane-producing microorganisms [146]. In addition to its properties, biochar can also improve the performance of a biogas digester by improving the quality of the feedstock [147].

The addition of appropriate additives can help to optimize biogas production and improve the efficiency of the anaerobic digestion process. Various other materials can be used as additives in anaerobic digestion for biogas production, aside from biochar; they include metal oxide nanoparticles [148,149,150] and chitosan biopolymer [151,152]. Biochar has several advantages over these materials in terms of cost, stability, adsorption capacity, habitats for microorganisms, and sustainability. However, the choice of material ultimately depends on the specific conditions of the anaerobic digestion process, the characteristics of the substrate being used, and the desired outcome [153].

### 2.5. Review of the Experimental Methods Used for Characterizing Biochar Molecular Properties

**Figure 1:**
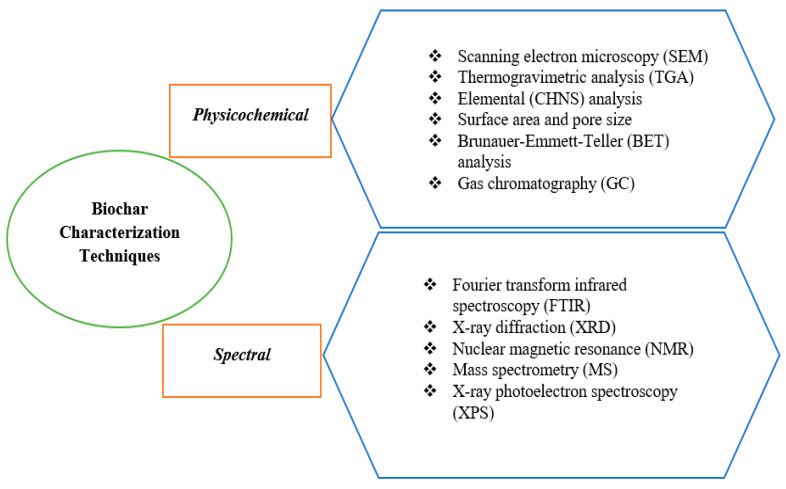
Commonly used techniques for biochar characterization.

To understand the properties of biochar and how they affect its performance in various applications, a wide range of experimental methods have been developed to characterize its molecular properties. Some of the commonly used experimental methods for characterizing biochar molecular properties and their limitations are discussed below. The characterization methods can be divided into physicochemical and spectral techniques, as seen in Figure 1. Some techniques, such as Scanning Electron Microscopy (SEM) and Thermogravimetric Analysis (TGA), can also provide spectral information, in addition to physical and chemical properties.

Fourier Transform Infrared Spectroscopy (FTIR) is a widely used technique for characterizing biochar’s molecular properties [154]. This technique measures the absorbance of infrared radiation by functional groups in the biochar [155], providing information about the chemical composition or functional groups within the biochar of the material, such as hydroxyl, carboxyl, amine, and lactonic and amide groups which are important for its reactivity and interaction with the environment [122,156]. However, FTIR has some limitations, including the fact that it only provides a qualitative analysis of functional groups [157] and cannot provide information about the spatial distribution of functional groups within biochar [158]. 

SEM is a technique that is commonly used to study the surface morphology and pore structure of biochars. This technique can provide information about the size, shape, texture, and distribution of pores within the biochar particles [159], which is important for understanding its properties and performance. However, SEM has some limitations, including the fact that it requires a high vacuum environment for optimal operation, which can affect the structure and composition of the biochar [160].

Brunauer-Emmett-Teller (BET) analysis is a widely used method for determining the specific surface area of biochars. The specific surface area is an important property of biochar, as it affects its ability to adsorb and retain nutrients, metals, and organic compounds. BET analysis is based on the measurement of the adsorption and desorption of a gas (usually nitrogen) on the surface of the biochar. The resulting isotherm can be used to calculate the specific surface area of the biochar. However, BET analysis has some limitations, including the fact that it assumes that the biochar has a uniform pore-size distribution, which may not always be the case [161]. For the adsorption of gas molecules to take place, this method takes a long time [162]. Pore size distribution (PSD) analysis is a method used to determine the size and distribution of pores in biochar. The pore size and PSD are important properties of biochar because they affect its ability to retain water and nutrients [163], as well as its gas exchange properties [164]. Gas adsorption/desorption is a widely used and powerful technique for determining the pore size distribution of a wide range of materials, including porous solids, such as zeolites, activated carbons, and metal-organic frameworks [165]. Gas adsorption/desorption is a technique similar to BET analysis but provides information on the distribution of pore sizes in biochar. Gas adsorption involves exposing a sample to a gas, typically nitrogen or argon, and measuring the amount of gas that is adsorbed onto the surface of the sample as a function of pressure. By analyzing the adsorption isotherm, which is a plot of the amount of gas adsorbed as a function of pressure, the pore size distribution of the sample can be determined. Several models can be used to analyze the gas adsorption isotherm, including the BET (Brunauer-Emmett-Teller), Langmuir, and DFT (Density Functional Theory) models [166,167]. However, this method of characterization has some limitations, including the fact that it only provides information about the size and distribution of pores, and not their shape or connectivity.

X-ray diffraction (XRD) is a technique that provides information on the degree of graphitization, crystal phase, and crystallinity of biochar [168,169], which is the advantage that it has over FTIR. XRD provides an understanding of the properties and behavior of biochar in various applications [170]. This method’s requirement for access to a standardized reference file of inorganic compounds (d-spacings, hkls) is one of its limitations [171].

TGA is a technique used to study the thermal stability and decomposition behavior of biochar [172]. TGA can provide information on the thermal properties of biochar, including the onset and completion temperatures of the different decomposition stages [173]. TGA’s main drawback is that degradant production does not always correlate with the mass loss of volatiles [174]. TGA instrumentation is more expensive than Loss of Ignition equipment by a factor of roughly four [175].

Gas Chromatography/Mass Spectrometry (GC/MS) is a technique used to identify and quantify the chemical compounds present in biochar [176]. GC is a physicochemical technique and MS, on the other hand, is a spectral technique. GC-MS can provide information on volatile and semi-volatile compounds, including polycyclic aromatic hydrocarbons (PAHs) and other organic compounds [177,178].

The amount of the elements contained in a material is usually determined as percentage content of total hydrogen, nitrogen, carbon, oxygen, and sulfur using elemental analyzers [179,180].

Nuclear Magnetic Resonance (NMR) is another commonly used powerful technique for characterizing biochar molecular properties. This technique provides information about the structure of organic molecules within the biochar, including the composition of biochar [181]. It can provide information on the distribution of carbon species in biochar, including the types of functional groups, the degree of aromaticity, and the extent of cross-linking (spatial distribution) [182]. However, NMR has some limitations, including the fact that it is a relatively expensive and time-consuming technique that requires specialized equipment [183].

X-ray Photoelectron Spectroscopy (XPS) is a surface-sensitive analytical technique used to obtain information about the chemical composition of a biochar’s surface layers [184]. XPS is an essential tool for characterizing and understanding materials at the atomic and molecular level [185]. XPS typically measures up to 1–10 nanometers in depth and may not provide information about the bulk properties or deeper layers of a sample [185,186]. 

These methods can provide a comprehensive understanding of the molecular properties of a biochar, which can be useful for optimizing their performance in various applications.

## 3. Negative Influence of Additive Carbon Material on Microorganisms in the Biogas Yield

**Figure 2:**
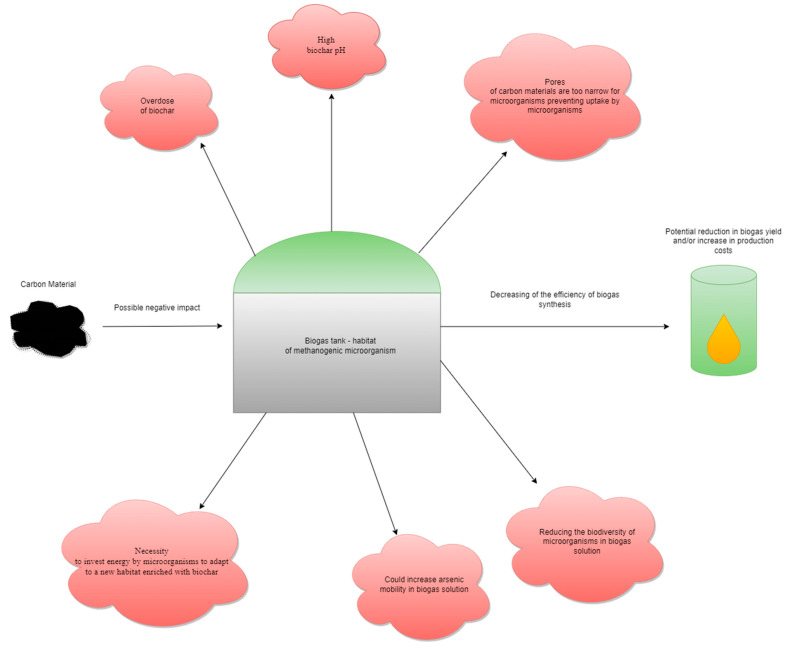
Examples of possible negative effects of biochar on anaerobic digestion.

A discussion of the negative influence of additive carbon materials on the performance of anaerobic digestion systems for biogas yields, such as reduced biogas yields, the inhibition of microbial activity, changes in the microbial community composition, and alterations to fermentation pathways, will be approximated in the following subsections. The authors of this publication will mainly focus on three carbon materials, biochar, hydrochar, and activated carbon. Examples of the possible adverse effects of biochar on anaerobic digestion are given in Figure 2.

### 3.1. Influence of Biochar on Changes in the Microbiocenosis Habitat and Colony Growth

The efficiency of adding biochar to anaerobic digestion microorganisms is highly dependent on the substrate used to make the biochar. For example, experiments conducted by [136,187], and [188] have proven that the use of different substrates of biochar highly changes the composition of microorganism species and even the phylum. It is worth adding that even the mineral particle ratio in biochar structures could be highly affected by the species composition during anaerobic digestion [189]. The influence of biochar on microbiomes was proven by [190]; moreover, [191] proved that biochar and carbon felt could increase the microbiome ratio of *Methanosaetaceae*.

The high sorption features of biochar could be positive because of pollutants absorbed during anaerobic digestion [192], but this feature could also be problematic. For example, when a methane fermentation solution has a low nitrogen ratio, an excessively large amount of biochar additives could lead to a deficiency it nitrogen and make it inaccessible to microorganisms [140,193]; moreover, a high pH of biochar could also be harmful for microbiocenosis [140]. Needless to say, the highly porous structure has some advantages, but, on the other hand, the small pores could trap some nutrients or elements inside, and because of their dimensions, prevent microorganisms from adsorbing those kinds of crucial elements [43]. In this case, the golden mean should be kept when designing the conditions for creating biochar to ensure the appropriate pore size for adsorbing pollutants and such that microorganisms have possible access to any nutrients that may be present in the pores. However, this could be a very difficult task, and perhaps it will be easier to control the content of the nutrients taking into account the possible “losses” of the nutrients in the pores.

The high pH of biochar could influence the pH level of the methane fermentation solution, and hence, the alkaline solution could start converting NH_4_ ions into toxic NH_3_, which could pose a potential threat to microbial communities [194]. Interestingly, some studies have found no connection between the influence of the physical properties (like electrical conductivity and surface area) of the biochar and an increase in the biogas yield, although it could increase the rate of anaerobic digestion [195].

That change could have a beneficial effect on biogas production because it could allow for the development of microorganism’s abundant species, but it could also disturb a microorganism balance in the methane fermentation solution habitat; for example, it could decrease beneficial microorganisms in the microbiome mix and/or increase the amount of unnecessary microorganisms. Moreover, changing the habitat features could force beneficial microorganisms to use some amount of energy to adapt to new environmental conditions [196]. Solutions with low concentrations of nickel generally promote two types in microbial communities, *Methanosarcina* and *Methanosaeta* [197]. Biochar itself could also have more negative effects on eubacteria (like *Firmicutes* and *Proteobacteria)* than archons [50].

Pyrochar produced in high-temperature pyrolysis (like 700 °C) could decrease the bacterial community from *Methanosaeta* species [198]. Biochar could also present a harmful influence on microorganisms by releasing toxic elements directly into an anaerobic digestion solution; for example, biochar modified by KH_2_PO_4_ could increase arsenic mobility in swine manure used as a biogas substrate [52]. This property of biochar, despite the quite good stabilization of heavy metals, such as Cr, Cu, Pb, and Zn, should be taken into account when trying to use waste containing high concentrations of heavy metals for energy purposes [199]. Further, it is worth paying attention to the feedstock used for the production of biochar, because if we want to use waste material by turning it into biochar directly created for carbon material additives, there may be a risk of releasing heavy metals from the biochar into the methane fermentation solution. This can be a significant challenge in the use of carbon additives to increase biogas yields in a circular economy.

It is worth noting that the very important aspects during the use of biochar-like additives in biogas production increase, as is commonly known “only the dose makes the poison”, and this sentence could also be accurate in this case. The overdose of biochar in an anaerobic digestion solution could negatively affect methanogenic efficiency and extend the lag phase [42].

### 3.2. Hydrochar Influence of Microbiocenosis during Methane Production 

Hydrochar is a type of carbon material that could be produced from wet material, like fruit pomace [200], kitchen waste [201], or sewage sediment [202], with the use of a hydrothermal carbonization process. Needless to say, that kind of process could help to utilize high-moisture waste and transform it into useful fuel. This kind of material could be used similarly to biochar (sometimes even with better results [42], and also as an additive for improving the biogas yield [202,203]. 

In general, hydrochar additives could improve biogas yields by promoting DIET and selecting microbiocenosis into a more productive mix with an increase in the *Methanobacterium* percentage [203]. Another noteworthy study [204] explores the use of hydrochar as an adsorbent for ammonia, a compound with potential biogas production benefits, in anaerobic digestion. However, the results indicate that the adsorption of ammonia by hydrochar may not significantly enhance biogas production. Moreover, hydrochar could enrich some microorganism species, like *Methanobacterium, Methanosaeta*, *Clostridium,* and *Methanosarcina* [205]. Unfortunately, hydrochar additives, of course, could enrich species, like *Methanosaeta* or *Syntrophomonas*, but also, at the same time, it could be harmful to the population of acidogenic and hydrolytic groups of microbiocenosis, for example, *Acinetobacter* [51].

But, adding hydrochar to an anaerobic digestion solution does not always improve biogas production [204,206]; the properly chosen ratio of hydrochar promotion is crucial for improving biogas production and supporting microbiomes. Another important factor during the use of hydrochar in improving biogas production yields could be the temperature during the HTC process, as in an experiment provided by Choe et al. [204]. Hydrochar usually improves the biogas yield, except in the case when a tofu residue was caused by hydrothermal pretreatment at a temperature of more than 140 °C when the biogas yield starts to decrease linearly [204]. Needless to say, the negative influence of hydrochar on microbiocenosis requires further research. 

### 3.3. Influence of Activated Carbon on Microbiocenosis during Methane Production

Activated carbon is a carbon material characterized by strong porosity, due to the large surface area [207]. That area is crucial for the most important feature; it could be a great chemical adsorbent. Using activated carbon during the anaerobic digestion process could increase biomethane production, very similar to adding biochar and hydrochar, which were described in previous acts. Moreover, adding activated carbon to a methane fermentation solution could increase a population of similar groups for example; *Methanosaeta* [61] and *Methanosarcina* [208]. Moreover, activated carbon could be used as an adsorbent to remove H_2_S from the biogas mix [209]. 

Activated carbon could reduce pathogenic microorganisms even by 18%, but on the other hand, this additive could harm microorganism biodiversity in the methane fermentation solution habitat [210]. It could be worth taking a closer look at the influence of decreasing biodiversity and its effect on the biogas production ratio. Quality and safety of the fertilizer from the biogas production process with activated carbon additives could be also interesting for further research.

### 3.4. Impact of Nanoparticles 

Nanoparticles are particles that do not exceed 100 nanometers but are larger than 1 nanometer [211]. Despite their small size, their influence on microorganisms is very significant; thus, metal nanomaterials from biochar have high levels of reactivity, a widespread surface area, and strong surface energy. Moreover, it is possible to modify the surface properties of biochar by using nanometal materials [54], that feature could be helpful in the adsorption of pollutants from a biogas tank solution. Needless to say, not even nanoparticles, like a part of the biochar component, could influence microbiocenosis, and even changing the size of biochar could change its properties. For example, the features of macro-size biochars are different from nano biochars [212]. 

Silver nanoparticles are strongly harmful to microorganisms [22], so it is very important to alleviate that impact. This kind of nanoparticle could be adsorbed by biochar [213] and allow microorganism colonies to grow in the easiest habitat. 

It is worth saying that not only non-organic nanoparticles could affect the biogas yield. Nanographen could also have a harmful effect on microbiocenosis (for example, *Methanosaeta*, *Lactococcus,* and *Anaerolinea)* during long-term exposition in 120 mg/L concentration of nanographene in a methane fermentation solution [214]. That graphene additive, but on a macro-scale, could also decrease the population of *Methanosaeta* [215]. Moreover, too high of a concentration of graphene in the methane fermentation solution could be harmful to anaerobic digestion [215].

The summary of some examples of the negative impacts of carbon material additives on microbiocenosis in an anaerobic digestion habitat is presented in Table 1.

## 4. Strategies for Mitigating the Negative Influence of Additive Carbon Materials

### 4.1. Mitigation of Negative Effects of Carbon Additives

There are several methods revised in the current literature that enable the mitigation of the negative effects of carbon additives. Processes are listed among those with a pivotal impact on the concern of monitoring and optimizing the anaerobic digestion process, with a significant emphasis on the optimal balance of nutrients and microbial viability. Overall, the key to mitigating the negative influence of carbon additives on the biogas yield is to carefully monitor and optimize the digestion process to maintain an optimal balance of nutrients and microbial activity.

#### 4.1.1. Optimal Dosage of Biochar Additives

The dosage of carbon additives has been proven to play a significant role in the process of anaerobic digestion [151]. Generally, it has been observed that biochar additions act in favor of biogas production, where treated samples have higher cumulative biogas generation than untreated ones. Digestion paths suggest that organic loads and reaction stability provided by an alkaline pH can positively alter biogas generation.

However, when it comes to the biogas yield itself, the amount of added carbon is thought to be far more important than the listed aspects. Rasapoor et al. [195] proved that the amount of biochar add-ons that are out of a certain scope can massively affect the methane potential. Biochar add-ons tested on the Organic Fraction of Municipal Solid Waste (OFMSW) were shown to be effective only within a certain scope of dosage. The k-value, which refers to the first-order model rate constant of biogas production of the control group was higher compared to some of the samples with added biochar. Ten grams per liter of poultry waste biochar additive, together with 10 g/L and 30 g/L of pine sawdust, decreased the k value. Moreover, among all tested samples, the k value decreased when the concentration of added biochar reached 30 g/L, compared to lower doses.

Fagbohungbe et al. [216] showed that infusions of biochar add-ons did not increase the total methane production. The dosage tested in the study (35 g of biochar per 1 liter of citrus peel used as a substrate) has shown no statistically significant difference between the control and non-control groups. The optimum dosage of carbon additives depends on the type of substrate, size, capacity of the digester, and other characteristics. The dosage of biochar should be determined through experimentation conditions and should be thoroughly reviewed.

Y. Shen et al. [217] showed that an unoptimized dosage of biochar addition paired with the effect of time digestion can negatively influence the CH_4_% in the anaerobic digestion process. The results of a statistical analysis disclosed that high dosages of biochar enhanced the process stability, but may have caused inhibitory effects. Microbial activity and process kinetics were described as suppressed, and the cumulative volume of biogas was lower than the control group.

In Sunyoto et al. [218], the cumulative methane production with the addition of biochar equal to 33.3 g/L was significantly lower (931.7 mL/L) than the cumulative methane production without any addition of biochar (1070.0). The highest noted methane production occurred at 8.3 g/L and equaled 1136.6 mL/L, but was not statistically significant from the control group. According to [219] and [220], where similar test results were attained, the reason behind this was high amounts of propionic acid. The energy needed to oxidize propionic acid to acetate was high, and its accumulation in the reactor resulted in a slower conversion of propionic acid at the acetogenic rate.

Optimize the dosage of carbon additives: The optimal dosage of carbon additives should be determined through experimentation to avoid overloading the system with carbon, which can lead to a decrease in the biogas yield. It is important to ensure that the ratio of carbon to organic matter in the feedstock is within a suitable range. Select appropriate carbon sources: The choice of carbon sources should be carefully considered to avoid the introduction of contaminants that could negatively impact the biogas yield. The carbon sources should be pre-treated or screened to remove any unwanted materials that could inhibit the digestion process.

#### 4.1.2. Pretreatment of Biochar (Surface Modification)

The concept of pre-treating biochar to obtain higher biogas yields has been extensively studied and discussed for the past 20 years. It has been proven that pre-treated biochar can positively affect the biogas output [210,221,222]. In Zhang et al. [210] biochar pretreated with zinc chloride, phosphoric acid, and potassium hydroxide exhibited more pores on the surface structure. Compared to the untreated sample, which had fewer well-developed pores and an overall smoother structure, pre-treated samples had a larger surface area due to high porosity.

The choice of pretreatment method depends on the specific characteristics of the biochar. The pretreatment of biochar can refer to a process used to modify its physical/chemical or biological properties to acquire additional features. Modifications can be particularly ascribed to surface modification, and those treatments can be divided into four main categories:

- Physical treatment;
- Thermal treatment;
- Chemical treatment;

Physical pretreatment involves grinding, sieving, or activating, and chemical treatment involves the direct use of chemical agents or substances to initiate oxidation, reduction, or impregnation. Thermal treatment relies directly on the use of temperature, and it is possible to perform it before and after biochar production. Microbial (or biological) treatment involves the use of microorganisms and can potentially enhance the biological activity and nutrient availability of microorganisms in the anaerobic digestion process. Proper pretreatment can improve the efficiency of the anaerobic digestion process and increase the yield of biogas.

##### Physical Treatment

There are physical methods that focus on biochar modification to obtain a material that is capable of removing contaminants, which can have an indirect impact on increasing biogas.

Carbon Dioxide Activation: Biochar is pyrolyzed and activated using carbon dioxide (CO_2_). The CO_2_ reacts on the surface of the biochar, forming surface oxides and increasing the physicochemical properties, like the microporosity, specific surface area, hydrophobicity, and aromaticity. Carbon dioxide treatment can adsorb sulfamethazine [223], copper [224] and a hypochlorite solution [225]. 

During the microwave modification, electromagnetic waves of a specific frequency are used to activate the biochar. This activation technique provides uniform and quick internal heating with the help of three mechanisms, which are ionic conduction, dipole polarization, and interfacial polarization. This process is different from conventional methods where heat is transferred from the surface to the material causing variations in the temperature and conduction phenomena. Microwave heating generates microplasma spots throughout the reaction mixture, which results in enhancing the process of the chemical reaction and its local temperature, leading to the activation of biochar [226].

##### Thermal Treatment/Thermal Activation

Biomass is thermally decomposed at high temperatures in a low-oxygen environment. This process involves the dehydration of biomass, followed by the decomposition of organic molecules, such as cellulose, hemicellulose, and lignin. The result is the transformation and rearrangement of the molecular structure of the biomass. There are various examples of the removal of contaminants with the use of biochar materials, i.e., the removal of molybdenum [227], polyaromatic hydrocarbons [228], and organic compounds [229].

##### Chemical Treatment

The solution proposed by M. Zhang et al. [230] was adding biochar-supported nanoscale zerovalent iron during anaerobic digestion at a mesophilic temperature. Enhancement effects were investigated for methane production and its impact on the microbial structure. The biochar and nanoscale zerovalent iron composite was effective in terms of enhancing process stability. Intermediate organic acids were generated and degraded more efficiently. Compared to the biochar composite with nanoscale zerovalent iron, pristine biochar had a higher carbon content and a lower O/C ratio. The composite contained more oxygen functional groups; hence, its adsorption abilities were positively altered [231]. In addition, nanoscale zerovalent iron can improve methane production, where Fe^+^ serves as an electron donor for reduction as it oxidizes under anoxic conditions [232,233].

Pairing nanoscale zerovalent iron with biochar is a relatively recent concept, although Fe add-ons have been already tested a few years back and gained popularity due to their influence. Results presented by [116] showed a novel strategy for accelerating hydrolysis. Acidogenic performances during chemical oxygen demand removal were not prone to the hydraulic retention time. Methanogenesis and acidogenesis were optimized as the propionate production was decreased [234].

Apart from nanoscale zerovalent iron, [192] proposed another solution to mitigate the negative effects of biochar add-ons and compared the results of pristine biochar and MnFe_2_O_4_-modified biochar. The study investigated the anaerobic digestion performance and CH_4_ production with the addition of corn straw biochar and manganese oxide oxide-modified biochar (MnFe_2_O_4_-biochar). The highest cumulative methane production occurred at 1.5 g of MnFe_2_O_4_-biochar per 1 g of dry matter of substrate and equaled 211.57 mL/g. The 0.5 g of pristine biochar per 1 g of dry matter of substrate resulted in 141.4957 mL/g, whereas the cumulative methane production for 0.5 g of MnFe_2_O_4_-biochar per 1 g of dry matter of substrate equaled 147.09 mL/g. In addition, the surface area of the biochar increased from 2.39 m^2^g^−1^ to 30.37 after modification with MnFe_2_O_4_. This can indicate that a more porous structure favored pollutant adsorption. It is important to note that further research and experimentation are needed to better understand the mechanisms behind this improvement and to assess its applicability under various conditions. Nevertheless, these findings suggest that modified biochar has the potential to be a more effective additive in anaerobic digestion processes than pristine biochar. Apart from that, an increased amount of *Methanosarcina* bacteria could potentially enhance the conversion of organic compounds, i.e., acetate into CH_4_.

An evaluation of the effects of modified biochar with ammonium hydroxide (NH_4_OH) and hematite nanoparticles (α-Fe_2_O_3_ NPs) proved that this carbon material could be an effective way to increase the methane yield [10]. The major positive effect was noted when the substrate was treated with 100 mg of sawdust biochar combined with 10 mg of α-Fe_2_O_3_ NPs, compared to the group treated with manure itself (219 mL/g vs. 138 mL/g). Although the biogas yield was higher when the substrate was treated with 100 mg of unmodified biochar, the yield was lower compared to that with modified biochar (205 mL/g vs. 219 mL/g).

Research by [235] on the addition of cerium chloride along with biochar resulted in a higher cumulative yield of CH_4_.The addition of both Ce^3+^ and biochar to the substrate caused the highest yield among all tested add-ons (biochar, Ce^3+^, and group with no add-on). The yield was higher by 4.4%, 5.7%, and 22.3%, respectively. Biochar and cerium chloride synergistically increased the production of methane. It was clear that biochar and cerium chloride did not reach the same cumulative yield as they did simultaneously. However, only the combination of both reduced the time required for hydrolysis and accelerated the degradation of organic compounds.

Palniandy et al. [222] showed that sodium hydroxide treatment altered the surface area, decreasing the porosity of biochar materials. Though the ash was removed from the biochar, the decomposed organic matter entered pores and blocked them, which resulted in a lower surface area.

Choudhury and Lansing [236] checked the influence of iron-impregnated biochar on H_2_S production during anaerobic digestion. Compared to the same dose of corn stover non-impregnated biochar, where removal efficiency was at 52%, Fe-biochar led to complete hydrogen sulfide removal.

The proper choice of the pretreatment method depends on the specific characteristics of biochar and the requirements of the anaerobic digestion, reactor, type of substrate, pH, and temperature. Selecting a pretreatment method must effectively augment biochar accessibility to microorganisms and minimize the energy requirement.

#### 4.1.3. Adsorbent Addition

Adsorbents can act as a medium that adheres to unfavorable compounds present during the anaerobic digestion process [237]. As biochar can act as an adsorbent itself, the mitigation of inhibitors of different origins can be carried out by zeolites, activated carbon, silica gel, and activated alumina [140,238]. Adsorbents can effectively mitigate heavy metals (Na^+^, K^+^, Ca^+^, Al^+3^, Cu^+2^, Zn^+2^, Cr^+3^, and Ni), organic compounds (chlorophenols, halogenated aliphatics), volatile fatty acids (VFAC), and ammonia [218,220,239].

Zeolites have been mainly used to remove ammonium and mitigate the negative effects of high nitrogen levels in reactors [240]. Zeolites are capable of exchanging cations (Ca^+^ and Mg^2+^) and the absorption of impurities and have catalytic abilities [241]. In Achi et al. [242], zeolite addition during anaerobic digestion resulted in the highest volatile solids (VSs) and chemical oxygen demand (COD) reduction. Moreover, the highest reduction in VSs and COD was in sync with the highest cumulative methane production [243,244]. Amalia et al. [245] showed that the longer zeolite purification of biogas resulted in higher methane contents in biogas.

Modified biochar can be used to remove compounds and immobilize CuO for efficient adsorption [246]. Biochar modified with potassium hydroxide (KOH) has a larger surface area and higher adsorption capacity than unmodified biochar [247,248] and pristine biochar [249].

#### 4.1.4. Appropriate Carbon Sources

To mitigate the negative effects of biochar during anaerobic digestion, it is essential to focus on materials that can balance the carbon-to-nitrogen (C/N) ratio [16,197,250]. Suitable carbon sources that are commonly used in anaerobic digestion systems are as follows:

- Food waste;
- Manure;
- Crop (agricultural) residues;
- Energy crops; and
- Microalgae biomass [16].

Rodriguez et al. [251] showed that biochar made of polyvinyl chlorine had higher ash and higher electric conductivity and could enhance methane production. Biochar prepared at 500 °C had a more suitable cation exchange capacity than the ones prepared at 600 °C and 700 °C. Poultry litter biochar had higher amounts of trace minerals, such as quartz and sylvite, and swine manure biochar had higher amounts of dolomite and sylvite, construction wood—dolomite and calcite, and pyrite and anhydrite.

Kizito et al. [252] showed that biochar adsorption efficiency is dependent on the material from which it was obtained. The adsorption of rice-husk-derived biochar was lower (39.8 mg/g) than biochar of wood origin (44.64 mg/g).

In Shanmugam et al. [253], among carbons of different origins, granular activated carbon resulted in the highest increase in CH_4_ (78%). Switchgrass biochar and Ashe Juniper biochar resulted in a 72% and 71% increase, respectively. Biochar made of holm oak residues resulted in only a 5% increase in the methane yield, and straw digestate biochar resulted in a 32% increase. On the other hand, granular activated carbon increased the chemical oxygen demand removal by 47%, whereas powdered activated carbon was about 108% more effective.

#### 4.1.5. Monitoring and Adjusting pH Levels

The effectiveness of an anaerobic digestion system predominantly depends on the pH value [35]. The drops in pH can hinder microbial activity and the microbial functioning of methanogenic bacteria [254]. To mitigate the negative effects of carbon additives during anaerobic digestion, it is important to maintain the pH within an optimal range of 6.5 to 7.5. This can be achieved by adding alkaline materials, such as sodium hydroxide or lime, or by using buffering agents, such as bicarbonate or phosphate. The alkaline materials can raise the pH by reacting with the acidic compounds in the digester, while buffering agents can resist pH changes by absorbing excess hydrogen ions (H+) or releasing H+ as needed to maintain a stable pH [255]. Alternative additives, such as trace minerals and enzymes, can be used in place of carbon additives to enhance biogas production. These additives can improve the activity of microorganisms and increase the breakdown of organic matter, resulting in higher biogas yields and optimized stability [256].

Heitkamp et al. [257] underlined the importance of a suitable pH inside a reactor, where the inhibition effect was reduced. The amount of propionic acid exceeding 1800 mg/L could inhibit the whole process of biogas production if the pH drops below 6.5. Higher pH corresponds to high NH_4_—4 contents, which provide buffering capacity and enable the biogas production to continue.

Carbon additives can alter the pH of the digester, which can negatively affect the activity of microorganisms responsible for biogas production. It is important to monitor and adjust the pH levels to maintain an optimal range for microbial activity [258].

A vital part of monitoring pH levels is to perform it regularly and adjust the amount of alkaline or buffering agents added as necessary to maintain the optimal pH range. By maintaining the proper pH levels, the negative effects of carbon additives can be minimized, and the anaerobic digestion process can be more efficient and effective [10].

#### 4.1.6. Use of Alternative Additives Blending Carbon with Other Materials

The reasons behind the lower methane yields during anaerobic digestion with biochar addition are described as the limitation of nutrients available for bacteria present during the anaerobic digestion process [8,259]. The absence of both macronutrients and micronutrients alters microorganism functioning on a basic level. A lack of trace elements will result in undernourished and starved bacteria that tend to be less effective in terms of digestion and interactions between different species present in the reactor [25,260]. To sustain constantly working bacterial groups through each pathway of the chemical reaction, additive materials shall be considered.

Phosphorus-rich materials include materials, such as bone meal, slaughterhouse waste, and microalgae. They provide phosphorus, which is an essential nutrient for microbial growth, metabolism, and the mitigation of ammonia inhibition [140,240].

Trace minerals, such as iron, zinc, manganese, cobalt, or selenium, are proven to positively alter the long-term digester stability, enhance organic matter degradation, and maintain relatively low volatile fatty acid levels [261,262] pointed out that the addition of TiO_2_ and ZnO/Ag powders can improve the biogas yield.

The choice of biochar additive depends on the specific requirements of the anaerobic digestion system and the characteristics of the biochar being used. It is essential to select an additive that is compatible with the anaerobic digestion system and provides the necessary nutrients and minerals for microbial growth and metabolism [263]. Alternative biochar additives can be used to enhance the performance of anaerobic digestion systems by providing essential nutrients and minerals to microorganisms. The proper selection and use of these additives can improve the efficiency of the anaerobic digestion process and increase the yield of biogas [264].

### 4.2. Consortia Adaptation

In anaerobic digestion, consortia adaptation refers to the process by which microbial communities adapt to changing environmental conditions and evolve during the process of biogas production. Microbial consortia are essential for the efficient functioning of anaerobic digestion systems. Alterations in the temperature, pH, and organic loading rates can result in changes in microbial communities. Maintaining stable conditions is crucial for optimizing continuous biogas production. 

Although various aforementioned researchers have established that char additives favoured biogas yields during anaerobic digestion, the processes and interactions between microorganisms are complex, as are the possible ways to alter them. Some reports described adaptation as a tool to increase microbial tolerance to shifts in conditions [201,265].

In Batta [266], tests between a previously adapted and non-adapted inoculum used in anaerobic digestion showed differences in biogas yields. The shift between samples showed that adapted microbial consortia produce more biogas than non-adapted consortia. The adapted batch had higher CH_4_ concentrations (92% more methane content). The adapted inoculum had a 106% increase in the methane content compared to the non-adapted inoculum. It has been shown that for previously adapted inocula, the microbial communities responsible for anaerobic digestion had different microbial communities than the non-adapted inocula.

The adaptation of consortia during anaerobic digestion occurs in response to changes in the feedstock or operational conditions [267]. For example, if the feedstock composition changes, the microbial community may adapt to utilize the new feedstock more efficiently [268]. Similarly, changes in the temperature, pH, or hydraulic retention time can also result in changes in the microbial community [269,270]. Overall, the adaptation of consortia during anaerobic digestion is essential for maintaining the stability and efficiency of the process. Understanding the dynamics of microbial communities and how they respond to changes in the environment is critical for optimizing anaerobic digestion systems.

## 5. Future Directions (Research Gaps) for Research and Applications

While there have been some reviews on the negative effects of biochar on biogas production and possible strategies to mitigate these effects, there are still several gaps in our understanding. The effect of functional groups on biogas production will depend on the specific type and number of functional groups present in the biochar, as well as the composition of the microbial community involved in the process. While there is some evidence that certain functional groups in biochar can have negative effects on biogas production, the mechanisms underlying these effects are not well understood. Further research is needed to better understand the complex interactions among the biochar, microorganisms, and environment in biogas production systems since there is a limited understanding of the mechanisms underlying the negative effects of biochar. The surface properties of biochar can be modified to improve its suitability for use in anaerobic digestion systems. Future research could focus on developing new technologies for modifying biochar properties, such as chemical treatments, thermal treatments, or nanotechnology-based methods.

Even though the addition of biochar to anaerobic digestion systems can improve biogas production, the optimal methods for adding biochar are not well understood. Further research is needed to evaluate the effects of different methods of biochar addition, such as mixing, layering, or co-digestion, on biogas production and system performance. Improving the management of anaerobic digestion systems can also help to reduce the negative impact of biochar on biogas production. This includes optimizing the feedstock composition, temperature, pH, and retention time, as well as implementing strategies for maintaining stable microbial communities.

The environmental impacts of using biochar in anaerobic digestion systems are not well understood. Overall, addressing these research gaps will be critical to advancing our understanding of the use of biochar in biogas production systems and developing effective strategies to reduce its negative impact on biogas production. This will also require a multi-disciplinary research approach that integrates knowledge from microbiology, engineering, and environmental science.

## 6. Conclusions

Anaerobic digestion is a complex process influenced by various factors, and the use of carbon materials, such as biochar, can have both positive and negative effects on biogas production. It is crucial to consider each case individually, accounting for potential interactions among different parameters. While many studies have shown that biochar can enhance biogas production, some instances demonstrate its adverse impact. These negative effects could be attributed to factors, like the release of heavy metals from certain types of biochar. This raises concerns, especially when dealing with organic waste from heavily polluted sources. To advance the implementation of a circular economy system, further extensive research on the potential drawbacks of biochar in anaerobic digestion is needed.

In practice, maintaining pH control after adding carbon materials and optimizing the dosage of additives appears to be crucial. Future research may pinpoint which carbon materials and production conditions are unsuitable for specific substrates, offering valuable guidance for biogas plant operations. In light of this review, there is a clear demand for more research to optimize biogas production by understanding the molecular properties of carbon materials and their interactions with microorganisms. Identifying and mitigating factors that hinder biogas yields will improve the efficiency of anaerobic digestion processes. This critical review underscores the significance of considering the molecular properties of carbon materials and their influence on microorganisms, paving the way for more effective biogas production.

## Figures and Tables

**Table 1:** Summary of some of the negative impacts of individual carbon material additives on microbiocenosis in an anaerobic digestion habitat.

Type of Carbon Material	Physicochemical Properties of Carbon Material	Potential Problem	Type of Negative Influence	References	Solution Proposal
Biochar	Strong porosity structure and adsorption capacity	Overdose of biochar	Scarcity of nitrogen supply for microbiocenosis when the ammonia nitrogen concentration is low	[42,140]	Care should be taken to choose the right dose for the biogas plant and remember that the dose should always be adjusted to the substrate used
Biochar	High biochar pH	High biochar pH that could promote the transformation of NH_4_^+^ into NH_3_	A high pH of biochar could promote the conversion of NH_4_^+^ to NH_3_, which could be harmful to the microbiocenosis during anaerobic digestion because NH_3_ is more toxic than NH_4_^+^	[140]	Monitor the pH level of the solution on an ongoing basis before and after adding biochar and correct the pH if necessary, depending on the possibilities
Biochar	Strong porosity structure	Pores of carbon materials are too narrow for microorganisms, preventing uptake by microorganisms	Too narrow pores could prevent absorbing nutrients and crucial chemical compounds for microbiocenosis	[43]	Preventing the formation of micropores that are inaccessible to microorganisms could be a difficult challenge. In this case, it is proposed to adjust the amount of medium, taking into account that some will be retained in the pores
Biochar/Hydrochar/Activated carbon/Graphene	Strong porosity structure and adsorption capacity, high biochar pH, a chemical component of the carbon material, and the content of heavy metals in the biochar. And any other properties that can affect the habitat of AD microbiocenosis.	Reducing the biodiversity of microorganisms in methane fermentation solution	Changing the properties of the anaerobic digestion solution that is a habitat for microbiocenosis may cause some groups of microorganisms to tolerate environmental change worse than others, which may disturb the original species composition.	[50,51]	Actions should depend on which bacterial species do not tolerate biochar additions. If these species do not participate directly or indirectly in the production of methane and the installation is industrial, this problem can probably be omitted or try to select biochar with other properties. Just remember to take the risk into account when modifying natural ecosystems with biocarbon additives
Biochar/probably most Carbon Material	Strong porosity structure and adsorption capacity, high biochar pH, a chemical component of the carbon material, and the content of heavy metals in the biochar. And any physicochemical property that may influence the change of the AD solution that is the habitat of the microbiocenosis	Necessity to invest energy by microorganisms to adapt to a new habitat enriched with biochar	Habitat changes may force microbiocenosis, part of energy expenditure, to adapt	[196]	The risk of whether biochar with given properties will be beneficial for microorganisms should be determined
Biochar	Adsorption properties of biochar	Could increase arsenic mobility in methane fermentation solution	Arsenic as a heavy metal could be harmful to methanogenic microorganisms	[51]	Choose a substrate with as few heavy metals as possible or try to use biochar with other properties. In addition, it is worth paying attention to the feature of feedstock from which it was used to create the biochar

## Data Availability

Not applicable.
